# Application of Translaryngeal Ultrasound (TLUS) in Patients with Neck Surgery—A Single-Centre, Prospective Cohort Study on Technique Evaluation

**DOI:** 10.3390/jcm11061691

**Published:** 2022-03-18

**Authors:** Sylwia Wolff, Adam Gałązka, Rafał Borkowski, Anna Gorzelnik, Marek Dedecjus

**Affiliations:** 1 Department of Endocrine Oncology and Nuclear Medicine, National Institute of Oncology, Roentgena 5 st., 02-781 Warsaw, Poland; sylviagajda@gmail.com (S.W.); borkora@poczta.onet.pl (R.B.); marek.dedecjus@gmail.com (M.D.); 2 Department of Head and Neck Cancer Clinic, National Institute of Oncology Maria Sklodowska-Curie Memorial Institute, Roentgena 5 st., 02-781 Warsaw, Poland; annagorzelnik@gmail.com

**Keywords:** translaryngeal ultrasound, thyroid surgery, vocal folds, thyroid carcinoma, recurrent laryngeal nerve

## Abstract

Purpose: The primary objective of this study was to assess the value of translaryngeal ultrasound (TLUS) in assessing vocal fold (VF) function in patients after thyroid, parathyroid and neck lymph node surgery. Methods: A total of 219 patients that underwent 230 surgical procedures were enrolled in this prospective study. The study was conducted from October 2020 to October 2021. Patients’ VFs were analysed independently with TLUS and laryngoscopy before and after the surgery. Various TLUS variables, such as vocal folds displacement velocity (VFDV), arytenoids symmetry and angle between VFs, were measured. The questionnaire evaluating discomfort caused to patients by both methods was conducted. Results: Of the 230 surgeries in this study, 85% were from oncological indications. The incidence of RLN injury was 10.4%. The accuracy of TLUS compared to laryngoscopy was 98.3%, with sensitivity 98.1%, specificity 100%, PPV 100% and NPV 83.3%. Laryngoscopy was found to cause significantly more discomfort than TLUS. VF visibility was lower in men; smokers; and patients with higher BMI (32 vs. 28 kg/m^2^), multifocal cancer, higher left lobe volume and higher fT3 levels. Arytenoid symmetry VFDV was lower for “e” and “i” right side and “i” left side in injured/disabled VFs/RLN. Conclusions: TLUS can be an excellent and non-invasive method of VF evaluation in most patients. There are some technical aspects that can improve its accuracy. Sometimes, RLN injury after the surgery, especially among oncological patients, is unavoidable. Therefore, it is vital to diagnose dysphonia early with convenient methods, such as TLUS.

## 1. Introduction

Ultrasonography, named a XXI century stethoscope, is a widely available, moderately cheap, and non-invasive diagnostic tool that is used in almost all specialisations [1]. Recurrent laryngeal nerve (RLN) injury and effectively vocal fold paralysis (VFP) are two of the most common complications of thyroid surgery (1–10%). RLN damage can also occur after parathyroid or lymph node removal. Complications of these procedures may be VFP, which means a complete inability to perform any movement, or paresis, which means a limited range of movements. RLN injuries can be divided into several groups depending on their duration: permanent or temporary, depending on the involved sides: unilateral or bilateral and depending on the position of the VFs: median, paramedian or lateral. Bilateral paralysis is a life-threatening situation, not rarely necessitating performing tracheostomy to enable breathing and avoid respiratory failure. A less serious consequence but still dramatically decreases life quality is voice impairment, which can present as problems with high pitch, hoarseness, change in sound and timbre of voice or difficulties with long speech [2,3,4]. Therefore, it is vital to assess VF function in all patients. The gold standard method is laryngoscopy. The proper technique and interpretation of this method require an additional tool (laryngoscope) and, most importantly, an ear nose and throat (ENT) specialist. It often causes discomfort for the patient, which can be managed with local lignocaine anaesthesia, which can induce an allergic reaction and be highly unpleasant. Laryngoscopy can also induce a gag reflex, be stressful and as an aerosol-generating procedure poses a threat of pathogen (e.g., SARS-CoV-2) transmission, which is especially important during a pandemic [5,6]. Ultrasound in patients with thyroid and parathyroid disease is a basic tool in the diagnostic process. It is possible to visualise VF transcutaneously with TLUS in most patients, and this technique can be routinely implemented during USG before any surgery. Only patients with invisible or dysfunctional VFs on a TLUS would be qualified for laryngoscopy. In effect, most patients could avoid additional, more unpleasant laryngoscopy. Moreover, the costs of ENT consultation and equipment would be limited and additional time could be saved [7,8,9].

The aim of the study was to assess the value of TLUS in assessing VF function in patients after thyroid, parathyroid and neck lymph node surgeries. Furthermore, this research aimed to answer the question of whether evaluation with TLUS will be possible among all patients or only in a selected group of individuals. What is more, the study analysed technical aspects of TLUS, determining which approaches and variables are most useful.

## 2. Materials and Methods

### 2.1. Design

This prospective study included patients after thyroid, parathyroid and neck lymph node surgeries. The study was conducted from October 2020 to October 2021. Surgeries were performed by a single team of 5 surgeons. Inclusion criteria comprised patient’s written consent and patients aged between 18 and 90 years. Exclusion criteria included larynx or trachea diseases, preoperative RLN injury (the exception were patients who had RLN damage at the first operation and they required another surgical intervention after some time), lack of consent, significant neoplastic infiltration of the larynx visible on the ultrasound or previous neck radiation.

### 2.2. Procedure Characteristics

**Figure 1:**
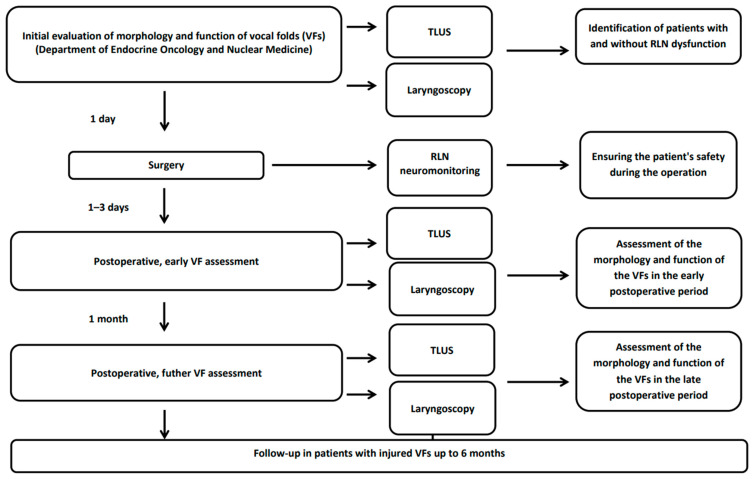
Scheme of the study. RLN—recurrent laryngeal nerve; TLUS—translaryngeal ultrasound; VF—vocal fold.

Ultrasound of the neck with simultaneous assessment of the vocal folds and laryngoscopy was performed 1 day before and 1–3 days after the procedure. Patients were evaluated independently using both approaches, with the physicians not being aware of the result of either the TLUS or laryngoscopy. In patients with VF dysfunction, follow-up was continued for up to 6 months (1, 3, 4 and 6 months after surgery). If the TLUS examination and the patient’s laryngoscopy on days 1–3 following the procedure revealed no abnormalities in the group of 128 patients, they likewise did not develop any deviation one month later (Figure 1). As a result, no additional follow-up was performed after 1 month in healthy individuals. TLUS was performed on the Philips Epiq with the eL 18–4 linear probe by the same physician. On the same day, a laryngoscope examination was performed by an ENT specialist.

For the sake of extreme caution and sterile conditions, the TLUS examination on days 1–3 after the procedure was performed with a sterile gel and a few centimetres above the dressing. During the ultrasound examination, the linear probe was placed in a transverse position over the front of the thyroid cartilage. If difficulty with visibility occurred, a lateral approach (probe placed transversely on the right and left side of thyroid cartilage) was attempted. The symmetry, mobility and position of VFs during breathing, coughing and phonation were also assessed. Paresis was seen as decreased movement of VFs and paralysis as a complete immobility. On the TLUS, we evaluated vestibular folds (“false vocal folds”), which lie above and slightly to the side of the true vocal folds and are not involved in voice formation. The laryngeal pocket has an entrance between the two pairs of folds. The vestibular folds on ultrasound are clearly visible as thicker, hyperechoic bands in the shape of an inverted letter “v”. In contrast, true vocal folds (the vocal folds that make up the glottis) are much thinner and more hyperechoic in TLUS and can only be visualised in some patients. In the ultrasound image, the movement of both types of folds is identical, i.e., by examining only the false VFs in the TLUS, we can infer that the true VFs have the same range of movement. The angle of inclination of the vocal folds from the midline was measured using both TLUS and laryngoscopy. One of the objective variables that was measured using TLUS was vocal fold displacement velocity (VFDV), which was measured via pulsed Doppler on a vibrating part of a vocal fold and expressed in cm/s. This parameter is proportional to the velocity of the wave causing vibrations of the vocal folds [10,11]. After vocal cord paralysis, this parameter should be significantly reduced. VFDV parameter was analysed while the patient was saying the vowels “a”, “e” and “i” separately [12]. It must be emphasized that patients were Poles and the Polish “a” is equivalent to an English “i”, “e” is “a” and “i” is “e”. Each vowel was measured a few times and its highest value was used for the analysis. Moreover, the position of the arytenoids, visible as hyperechoic, rounded structures located below the false VFs was assessed. An additional variable assessed using TLUS was the occurrence of the Doppler wave in a “crescendo–decrescendo” pattern.

Laryngoscopies were performed under local anaesthesia with 2% lignocaine spray if required. A Fiegert Endotech 70° oral endoscope was used in all cases. A standardised exam sheet was applied for vocal fold movement and position assessment. In addition, the examined patients were assessed using a visual analogue scale (VAS) from 0–10 to determine how much discomfort was caused by each of the applied research techniques. A score of 0 meant no discomfort and a score of 10 was equal to unbearable pain.

During the surgery, all patients underwent RLN and vagus nerve neuromonitoring to assess the signal and allow for a safe procedure.

Written informed consent to participate in the study was collected from each project participant. The study received approval from the Bioethics Committee.

### 2.3. Statistical Analysis

Statistical analysis was carried out in the R program, version 4.0.5. Nominal variables were described as frequency measures (count *n* and percentage frequency), and quantitative variables were described as frequency measures, central tendency and dispersion. The normality of the distribution was assessed using the Shapiro–Wilk test, based on skewness and kurtosis indices, as well as the visual assessment of the histograms. The relationship between the nominal variables was analyzed using the chi-square test or the exact Fisher’s test. The analysis of quantitative variables included the Student’s *t*-test for independent measurements and the Student’s *t*-test for pairs or their non-parametric equivalents, according to the fulfilment of the assumptions. TLUS assessment vs. laryngoscopy was performed by calculating the indicators of sensitivity, specificity, accuracy, NPV (negative predictive value) and PPV (positive predictive value). Logistic regression analysis was also used to establish predictors of nerve damage.

## 3. Results

### 3.1. Baseline Characteristics

A total of 219 patients from the National Institute of Oncology (NIO) in Warsaw were included in a prospective cohort study, including 49 men and 170 females. In total, 230 surgeries were performed by a single surgical team. The number of all patients (*n* = 219) was lower than the number of surgeries (*n* = 230), as some patients had more than 1 procedure. A total of 71 (32%) patients had obesity (body mass index (BMI) ≥ 30 kg/m^2^), 64 (29.2%) had hypertension and 36 (16.4%) had a history of nicotinism. Detailed baseline characteristics are presented in Table 1.

A total of 196 procedures were performed due to thyroid cancer suspicion or previously confirmed cancer. The most common procedures were lobectomy (*n* = 124, 53.9%) and thyroidectomy with central lymph nodes excision (*n* = 58, 25.2%). Forty-six (20.2%) surgeries were radicalisation procedures. In our group, 17 (7.4%) secondary surgeries (on the side of the neck previously operated on) were performed (Table 2).

In the histopathological report, there were 149 thyroid cancers confirmed; most of them were papillary cancers (*n* = 102, 68.5%). Metastases to the lymph nodes were found in 36 patients (15.7%), including 15 (6.5%) in the central nodes (N1a) and 21 (9.1%) in the lateral (N1b) lymph nodes. Among the 80 (34.8%) benign lesions, there were 61 (76.3%) cases without evidence of cancerous tissue (most of them were patients undergoing radicalisation contralateral lobe surgery after confirmation of a malignant tumor in a previously operated lobe). Among the other cases, 19 (23.7%) parathyroid adenomas were confirmed (Table 2).

**Figure 2:**
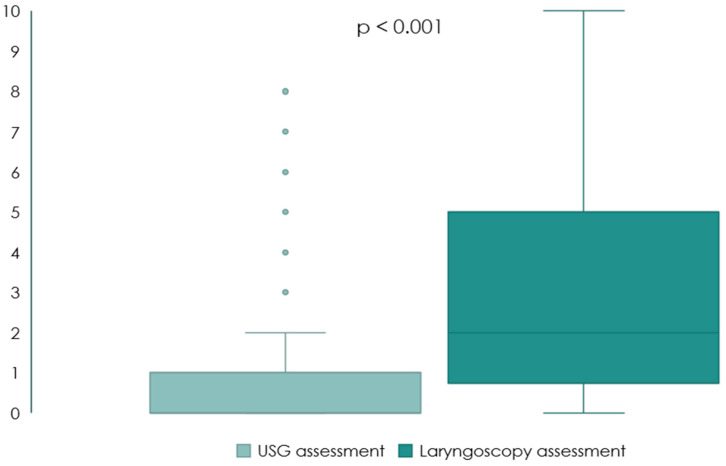
Comparison of TLUS and laryngoscopy in terms of discomfort on the VAS scale (*n* = 230).

Most patients (*n* = 150, 65.2%) stated that the laryngoscopic examination was much more uncomfortable compared to TLUS, with significantly higher points on the VAS, *p* < 0.001 (Table 2, Figure 2).

Detailed characteristics of the studied cases are included in the Supplementary Materials (Table S1, Figure S1).

### 3.2. Ultrasonographic and Laryngoscopic Findings

Table 3 presents data on the TLUS and laryngoscopy results from before surgery and follow-ups up to 6 months in patients with an RLN injury. Passive VF observations during different manoeuvres, VFDV values and angles were assessed using TLUS. Laryngoscopy evaluated the VF mobility, symmetry and rima glottis width. In addition, clinical voice changes were determined. There were 100% correlations regarding the VF subjective observations while the patient was either talking, swallowing or coughing. Subjective observation found that the best visibility of the VFs was obtained when the patient was whispering the “e”, “e”, “e” sound and the “ihi”, “aha”, “ehe” syllables.

### 3.3. Vocal Folds Displacement Velocity

VFDV values presented before surgery for the vowel “a” were around 140–141 cm/s, higher for “e”—147 cm/s and even higher for vowel “i”—150 cm/s. Stronger VFDV decreases on the right side for the vowels “e” and “i” and for vowel “i” on the left side were found in people with RLN dysfunction 1–3 days after the surgery (Table 4).

### 3.4. TLUS and Laryngoscopy Results

Table 5 provides accurate data on the sensitivity, specificity, accuracy, positive predictive value (PPV) and negative predictive value (NPV) of the TLUS compared to laryngoscopy. Based on the conducted research, the accuracy of the TLUS test reached a total of 98.3%, with 98.3% in women and 100% in men. PPV was 100%, while NPV was predictive of 83.3%. Slightly lower NPV values were observed in patients with BMI > 27.7 (78.6%) and body weight > 76 kg (76.5%) and in those <46 years of age (76.9%).

### 3.5. Vocal Fold Detection

Regarding the VF detection rate in the whole group (94%), it was possible to visualise VFs in a substantially higher number of women (82.0%) than men (7.7%). We also observed that invisible VFs were more often noted in patients who were smokers and had higher BMI (32.34 vs. 27.65 kg/m^2^), secondary surgery, multifocal cancer, T2 vs. T1b, higher left lobe volume and higher free T3 levels (Table 6). After surgery additional significant variable was calcium level which was higher among patients with invisible laryngeal structures. Considering the age criterion, the availability of VFs in ultrasound did not differ significantly (Figures S2 and S3).

### 3.6. Recurrent Laryngeal Nerve Injuries

There were 24 (10.4%) RLN injuries among all surgeries: 4 (1.7%) permanent and 20 (8.7%) transients. Vocal cord paralysis occurred in 16 (7%), and paresis in 8 (3.5%) patients with VF dysfunction. RLN damage occurred in 11.7% of patients with suspected or confirmed thyroid cancer, in 5.2% of patients with parathyroid adenoma and in none with mild thyroid disease. Among the RLN injuries, 95.8% were patients operated on for thyroid cancer, 4.2% for hyperparathyroidism and 0% for mild thyroid disease. The frequency of permanent RLN dysfunction in these groups was 17.4%, 0% and 0%, respectively, while the frequency of temporary injury was 87%, 100% and 0%, respectively. Comparison of findings in patients with dysfunction and healthy patients are included in Table 7.

**Figure 3:**
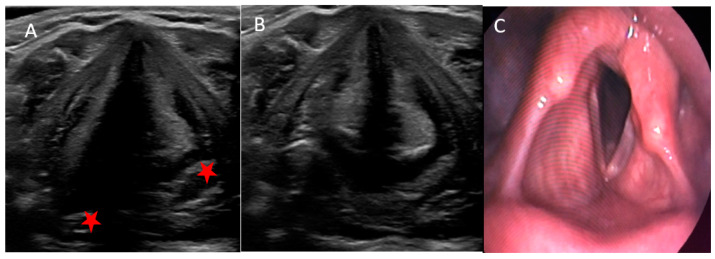
An example of left-sided paralysis, visible asymmetry of VFs and arytenoids (red star) and lack of movement of the left-hand VFs in adduction and abduction in TLUS (**A**,**B**) and during the videolaryngoscopy (**C**).

RLN function returned to normal after 3.5 months on average. Neuromonitoring of the vagus nerve revealed a decrease or loss of amplitude in 70.8% of injured RLN compared to 2.4% among intact nerves. We observed recurrent laryngeal nerve entrapment in excised lesions in 33% of patients with VF dysfunction. Besides TLUS (Figure 3A,B), some patients with RLN injury had also videolaryngoscopy to obtain exact, live images and movies of their VFs (Figure 3C).

## 4. Discussion

The number of thyroid and parathyroid surgeries increased in previous years and, therefore, the number of complications, such as vocal folds paralysis. Even if only a few percent of patients experience RLN injury, there are still a few thousand patients each year with voice impairment. Normally, less than 19% of thyroidectomies are performed due oncological indications [13]. In our group, it was the opposite, where only 15% were benign causes. This fact may explain a slightly higher number of RLN lesions in the study group because oncological procedures are usually longer, technically more difficult, the mass of the tumor may be massive and infiltrate the surrounding structures and, last but not least, some patients were operated on several times.

Our results are in line with the literature, as TLUS accuracy and sensitivity when compared to laryngoscopy was 98% in all cases, and specificity was even higher at 100%. We refer to the largest meta-analysis on this topic published in 2021 by Patel et al. in *J Clin Med* based on 16 prospective studies and 3332 patients. The authors of the study emphasized that TLUS, as a non-invasive, painless, well-tolerated, cost-effective and time-saving method, can be an effective diagnostic tool, especially in women and younger people. Ultrasonography can help to avoid unnecessary, invasive and potentially painful and uncomfortable laryngoscopy in approximately 80% of cases. The visibility of VFs before surgery was higher in our group before surgery at 94.3% vs. 86.3% and similar postoperatively at 93.5% vs. 94% compared to the presented meta-analysis results. The sensitivity and specificity of TLUS in the postoperative period (98.1% and 100%, respectively) exceeded the values presented in the meta-analysis of 84% and 96%. Interestingly, the diagnostic accuracy of TLUS was higher when the examination was performed by an operating surgeon or anesthesiologist compared to radiologists. In our study, TLUS was performed by the same endocrinology resident (Wolff) [14].

TLUS sensitivity ranged from 33 to 100% in different studies [12]. For instance, in a large study on 1000 patients, Wong et al., showed that TLUS is highly accurate regarding proper recognition of VFs dysfunction [15].

It was possible to visualise VFs in 94% of all cases, with 99% in women and 76% in men. Interestingly similar results were presented by Borel et al. in 2016, with 50% accessibility in men and 95% in women [7]. Carnerio-Pla et al. showed very unsatisfying visibility for men, with a mere 17% as opposed to 83% in women [16]. Thyroid cartilage begins to ossify in males at the age of 25 and can be entirely converted to bone by the age of 65. Thyroid cartilage never entirely ossifies in females and ultrasound waves can be transmitted easily in contrast to being reflected off bone [17]. In men with invisible VFs on TLUS with midline probe position, a lateral probe position should be attempted. In our group, this approach enabled visualising the larynx in an additional 21 patients (35% males with previously unseen VFs). This technique was described in the Atlas of Head and Neck Ultrasound 9 years ago [18]. The lateral approach was shown to be highly effective in a study from 2021 by Fung et al. The authors visualized VFs using this method in 93.3% of patients. This is a high percentage compared to the midline position of the probe (82.2%), and as in our results, this technique was particularly useful in men [19]. In a recent study by Knyazeva et al., it was shown that a gel pad significantly improves VF visualisation in male patients [9]. In the near future, we are planning to explore this technique.

VFs were possible to visualise on TLUS in significantly fewer overweight individuals compared to the normal weight group. Comparable conclusions were drawn by Kandil et al. [20]. We believe that the ultrasound beam is dampened by excess body fat in people with abnormal body weight. Age did not influence the TLUS visibility. Wong et al. presented results that also revealed this relationship for people over 70 years old [21]. As mentioned previously, this is the result of thyroid cartilage ossification. Other factors that influenced VFs visibility on TLUS with statistical significance were smoking, secondary surgery, tumor advancement along TNM scale, cancer multifocality, left lobe volume and free T3 levels, which have so far not been described in the literature.

Interestingly, in one person with a massive tumor measuring 92 mm, we observed very low VFDV and no VF visualization on one side. These values returned to normal after surgery, which resulted from the compression of the VF by the tumor mass. In the histopathological report, the lesion was found to be widely invasive thyroid cancer. This underlines the fact that TLUS is essential not only after surgery but also before the procedure. We were able to visualise one-quarter of the true vocal folds using TLUS. In the future, we should seek methods that improve true vocal fold visibility.

Postoperative arytenoids asymmetry in TLUS was observed in 83% of patients with RLN injuries, and in all individuals with RLN damage who were correctly diagnosed using TLUS. However, with the vocal cords intact, no asymmetry in their position was observed. The crescendo–decrescendo wave was more often observed in patients with RLN dysfunction, similarly to hoarseness and problems with a high pitch. The VFDV was extensively investigated in many studies and the results were very promising regarding the practical implementation of this objective parameter. Dedecjus, Dubey and Kumar showed that the VFDV was significantly lower (<50% or <40 cm/s or 60 cm/s) after surgery resulting in VF injury [11,22,23]. In the literature, normal values ranged from 60–300 cm/s [12]. In our study, the mean VFDV was highest for the vowel “i” (150 cm/s), slightly lower for “e” (147 cm/s) and lowest for “a” (141 cm/s). The VFDV change after surgery was significant for the vowels “e” and “i” for the right side and “i” for the left side; therefore, we recommend using the vowel “i” for assessment as the best of all studied sounds. VFDV was assessed when the patient was asked to say different vowels, such as “a”, “e” and “i”; this approach has never been described in the literature, where in other papers, patients were muttering only one vowel.

Using the Doppler method, the wave of vocal folds displacement during phonation resembling the shape of “crescendo–decrescendo” (with increasing and then decreasing amplitude) occurred in 17% in patients with RLN damage and in none with intact vocal cords. This indicated that this variable was somewhat specific, but not sensitive. In fact, these patients had a problem with longer speech, and their voices increased in intensity for a moment and then completely disappeared due to post-operative damage. This wave pattern in the TLUS was described by us for the first time in the literature. Moreover, if abnormal VF movement was visible on the ultrasound, TLUS correctly differentiated paralysis and paresis in almost all cases (95%). This fact has never been described in the literature before.

Postoperative vagus nerve stimulation is the most sensitive way to assess RLN function. The correct signal excludes the possibility of possible damage to the RLN in its course (from its departure from the vagus nerve to the entrance to the larynx) [24]. Neuromonitoring of the vagus nerve revealed a decrease or loss of amplitude during most surgeries with RLN impairment. It demonstrated that neuromonitoring is a highly effective method of intraoperative RLN function assessment. In the event of an injury, surgery on the opposite side should be postponed to avoid life-threatening bilateral paralysis, and in our study, we did not have any case of bilateral RLN palsy.

In our study for the first time, TLUS and laryngoscopy were compared in terms of patients’ discomfort. Most patients (65%) stated that the laryngoscopy examination was way more unpleasant than TLUS, while only 4% stated the opposite. Laryngoscopy is often associated with pain when pulling the tongue, and it is difficult to phonate or breathe during the examination. These results are another argument for the implementation of less invasive and more comfortable ultrasound assessments of patients.

The importance of proper RLN protection during surgery, early recognition of complications and prompt referral to an otolaryngologist were summarized in guidelines from 2013. All clinics should follow the protocol to provide the best standard care for all patients [25].

There are still some issues requiring thorough investigation. One example could be to search for the possibility of ultrasound evaluation of the external branch of the superior laryngeal nerve. Thus far, there is no data on how to visualise the function of this nerve using TLUS, which is responsible for high tone emission and modulates the timbre of the voice [26].

Finally, during the COVID-19 pandemic, it is especially crucial to introduce safer procedures, such as ultrasonography, that do not generate aerosol, unlike high-risk laryngoscopy. This aspect was appreciated in the latest publications and is another argument in favour of TLUS in most patients, with laryngoscopy being reserved for only disabled and invisible VFs [27,28].

## 5. Conclusions

TLUS can be a facilitating method of patient’s qualification to laryngoscopy after surgery. In this study, the results showed that in a vast majority of all patients, TLUS could be the only method of VF assessment required after surgery, while in a minority (with inaccessible or injured VFs or severe hoarseness), additional ENT consultation would be necessary.

This is the first study that analysed such a diverse range of variables that can be used to improve TLUS reliability. The obtained results showed that the movement of VFs are best assessed during whispering the vowel “e”. The vasalva manoeuvre, swallowing or coughing are not vital, but can be additionally implemented. VFDV can be useful if the value is lowered or shows a “crescendo–decrescendo” Doppler wave pattern, but it is not a necessary component of the TLUS evaluation and will not replace the observation of VF movement during phonation and respiration. Normal VFDV values can be found in patients both with and without RLN damage. Arytenoid’s symmetry is a highly accurate variable regarding distinguishing healthy and defective VFs and, in this study, its role in TLUS was assessed for the first time in the literature. Similarly, a crescendo–decrescendo Doppler wave pattern was assessed during TLUS for the first time. In individuals who present difficulty regarding visibility on TLUS, a lateral approach should be attempted because it allows for the visualization of VFs in a significant percentage of the subjects. Moreover, the added value was an implemented discomfort scale comparing the two methods. Additionally, for the first time we presented that the visibility of vocal cords during TLUS is significantly influenced by smoking, tumor size according to the TNM classification, tumor multifocality, left thyroid lobe volume, fT3 level and calcium level after surgery.

In the future, we plan to implement TLUS in routine neck ultrasound protocol; laryngoscopy would then be performed only among individuals with inaccessible or dysfunctional VFs. This would save money and time involved in ENT specialist consultation. Moreover, TLUS is a significantly more comfortable examination method for the patient. What is even more important, laryngoscopy is an aerosol-producing procedure that poses a great threat to medical personnel during a pandemic when compared to much safer TLUS. Early recognition of VFP is crucial for undertaking phoniatric rehabilitation and facilitates the return of a normal voice.

TLUS is a highly accurate, non-invasive, painless and quick method of vocal folds assessment. It can be used before and after neck surgery during a routine neck ultrasound scan. It is especially important to implement new, convenient techniques given the increasing number of thyroid and parathyroid surgeries. There is still potential for improving the TLUS technique, especially in males. There were some limitations of this study; for instance, variables such as VFDV, passive VFs observation, arytenoid symmetry lack standardisation and results varied between papers. The included group of patients was biased, as a vast majority of them were operated on due to oncological indications and those surgeries are more aggressive, invasive and burdensome than those for benign indications.

In conclusion, the study demonstrated that TLUS can be a great method of VFs assessment in most patients, and to make it more sensitive and specific, adjusting the visibility by different manoeuvres and approaches is crucial. We expect it will be more widely applied among physicians performing ultrasound, such as surgeons or endocrinologists. Thus far, TLUS cannot fully replace gold standard laryngoscopy, as it fails to properly diagnose all patients. Hopefully, increasing interest in ultrasonography will encourage doctors to transcutaneously assess VFs using this fascinating and simple technique.

## Figures and Tables

**Table 1:** Demographic characteristics of the patients (*n* = 219).

Variable	Level
Number of patients/surgeries	219/230
Sex, female, *n* (%)	170 (77.6)
Age, years, mean ± SD (*n* = 218)	48.31 ± 15.48
Height, m, mean ± SD	1.67 ± 0.09
Weight, kg, mean ± SD	78.46 ± 19.37
BMI, mean ± SD	27.92 ± 6.21
BMI level, *n* (%)	
Nonoverweight (BMI < 25.0 kg/m^2^)	74 (33.8)
Overweight (BMI 25.0–29.9 kg/m^2^)	74 (33.8)
Obese (BMI ≥ 30 kg/m^2^)	71 (32.4)
Smoking, *n* (%)	36 (16.4)
Asthma/COPD, *n* (%)	8 (3.7)
Hypertension, *n* (%)	64 (29.2)
Stroke/TIA, *n* (%)	-
DM/diabetes, *n* (%)	27 (12.3)

BMI—body mass index; COPD—chronic obstructive pulmonary disease; DM—diabetes mellitus; SD—standard deviation; TIA—transient ischemic attack.

**Table 2:** Cases’ characteristics.

Variable	*N*	Level
Number of surgeries	230	
Dysfunction, *n* (% of group)/% of all dysfunctions	230	24 (10.4)/100.0
Paresis		8 (3.5)/33.3
Paralysis		16 (7.0)/66.7
Reoperation, *n* (%)	230	14 (6.1)
BACC category, *n* (%)	192	
1		1 (0.5)
2		19 (9.9)
3		14 (7.3)
4		40 (20.8)
5		46 (24.0)
6		39 (20.3)
7—parathyroid		17 (8.9)
8—local cancer recurrence or lymph node metastases		16 (8.3)
Cancerous indication, *n* (%)	230	
Yes		196 (85.2)
No		34 (14.8)
Surgery indication, *n* (%)	230	
Thyroid carcinoma suspicion		196 (85.2)
Benign thyroid disease		18 (7.8)
Parathyroid disease		19 (8.3)
Type of surgery, *n* (%)	230	
Thyroidectomy with central lymphadenectomy		58 (25.2)
Hemithyroidectomy with central lymphadenectomy		124 (53.9)
Thyroidectomy with lateral neck dissection		15 (6.5)
Lymph nodes metastases surgery		12 (5.2)
Surgery of recurrent disease in postoperative bed		5 (2.2)
Isthmectomy		3 (1.3)
Parathyroidectomy		19 (8.3)
Trachea release		1 (0.4)
Secondary surgery, *n* (%)	230	17 (7.4)
Lesion location, *n* (%)	215	
Left		72 (33.5)
Right		86 (40.0)
Both		16 (7.5)
Parathyroid		20 (9.3)
Isthmus		9 (4.2)
Lymph node		13 (6.0)
Multifocal, *n* (%)	230	26 (11.3)
Lymph nodes metastases, *n* (%)	230	36 (15.7)
Lateral		21 (9.1)
Central		15 (6.5)
*N*, *n* (%)	129	
0	129	93 (72.1)
1a		15 (11.6)
1b		21 (16.3)
Cancer/benign on hist-pat, *n* (%)	230	
Benign		80 (34.8)
Cancer		150 (65.2)
Type of cancer, *n* (%)	168	
Papillary		102 (60.7)
Folliculary		11 (6.5)
Medullary		16 (9.5)
Hurthle		4 (2.4)
Anaplastic		1 (0.6)
Other cancer		4 (2.4)
Parathyroid		19 (11.3)
Border-line tumors group 1		11 (6.5)
Unpleasant USG, 0–10 scale, median (Q1; Q3)	230	0.00 (0.00; 1.00)
Unpleasant laryngoscopy, 0–10 scale, median (Q1; Q3)	230	2.00 (1.00; 5.00)
USG vs. laryngoscopy 1st assessment, *n* (%)	230	
More unpleasant laryngoscopy		150 (65.2)
More unpleasant USG		10 (4.3)
Both the same		70 (30.4)

Q—quartile; RLN—recurrent laryngeal nerve; SD—standard deviation; TSH—thyroid-stimulating hormone; WBC—white blood cells.

**Table 3:** Laryngoscopy and USG results in the total study group.

Variable	Before Surgery	1–3 Days after Surgery	1 Month after Surgery	3 Months after Surgery	4 Months after Surgery	6 Months after Surgery
*N*	230	230	128	13	8	8
Visible vocal folds	216 (94.3)	215 (93.5)	125 (97.7)	13 (100.0)	8 (100.0)	8 (100.0)
Normal vocal fold movement *	212 (98.1)	195 (90.7)	103 (83.7)	8 (61.5)	5 (62.5)	4 (50.0)
USG vocal fold symmetry still *	204 (94.4)	194 (90.2)	102 (83.6)	8 (61.5)	5 (62.5)	4 (50.0)
Symmetry during phonation *	204 (94.4)	194 (90.2)	102 (83.6)	8 (61.5)	5 (62.5)	4 (50.0)
Arytenoid symmetry *	203 (94.0)	194 (90.2)	103 (84.4)	8 (61.5)	5 (62.5)	4 (50.0)
Crescendo–decresceno wave *	No data	4 (1.9)	4 (3.2)	2 (15.4)	1 (12.5)	No data
Impaired vocal fold on USG						
Paralysis		15 (7.0)	15 (6.6)	4 (30.8)	2 (25.0)	2 (25.0)
Paresis		5 (2.3)	5 (2.2)	1 (7.7)	1 (12.5)	2 (25.0)
Proper function		194 (90.7)	194 (84.7)	8 (61.5)	5 (62.5)	4 (50.0)
Dysfunction seen in VFDV decrease		-	-	-	-	-
Invisible		-	15 (6.6)	-	-	-
Total angle	32.71 ± 4.29	32.47 ± 4.45	33.67 ± 5.71	31.54 ± 3.38	35.00 ± 6.14	32.50 ± 4.24
Laryngoscopy R mobility						
Normal	230 (100.0)	218 (95.2)	48 (81.4)	9 (69.2)	7 (87.5)	6 (75.0)
Paresis	-	5 (2.2)	6 (10.2)	1 (7.7)	-	1 (12.5)
Paralysis	-	6 (2.6)	5 (8.5)	3 (23.1)	1 (12.5)	1 (12.5)
Laryngoscopy L mobility						
Normal	230 (100.0)	217 (94.3)	49 (84.5)	11 (91.7)	6 (75.0)	6 (75.0)
Paresis	-	3 (1.3)	6 (10.3)	-	1 (12.5)	-
Paralysis	-	10 (4.3)	3 (5.2)	1 (8.3)	1 (12.5)	2 (25.0)
Rima glottis width laryngoscope	7.22 ± 0.77	6.92 ± 0.96	6.88 ± 1.12	7.54 ± 2.76	7.00 ± 1.07	6.88 ± 0.64
Vocal folds angle laryngosope	26.56 ± 3.87	25.44 ± 4.57	24.62 ± 6.11	24.00 ± 7.56	24.75 ± 4.77	20.75 ± 5.39
Hoarseness		57 (25.0)	18 (14.5)	3 (25.0)	2 (25.0)	1 (12.5)
Problems with high pitch		37 (16.2)	19 (17.3)	5 (41.7)	3 (37.5)	2 (25.0)
Dysphagia		4 (1.8)	2 (1.9)			

Data presented as *n* (%) for nominal variables or mean ± SD for continuous variables. *—% calculated relative to the number of cases with visible vocal folds.

**Table 4:** Vocal fold displacement velocity between healthy and dysfunctional groups.

	Dysfunction (cm/s)	Healthy (cm/s)	MD (95% CI)	*p*
Before surgery				
VFDV right a	137.19 ± 23.40	140.58 ± 24.86	−3.39 (−14.03; 7.24)	0.518
VFDV left a	138.98 ± 27.19	141.67 ± 22.41	−2.69 (−14.81; 9.43)	0.652
VFDV right e	151.74 ± 25.22	146.66 ± 23.21	5.08 (−6.24; 16.41)	0.365
VFDV left e	147.67 ± 27.36	147.34 ± 22.91	0.33 (−11.87; 12.54)	0.955
VFDV right i	151.39 ± 23.35	149.46 ± 23.95	1.93 (−8.65; 12.51)	0.711
VFDV left i	153.14 ± 26.05	150.85 ± 22.10	2.29 (−9.34; 13.93)	0.689
1–3 days after surgery				
VFDV right a	120.40 ± 39.57	138.70 ± 23.16	−18.30 (−35.68; −0.92)	0.040
VFDV left a	130.48 ± 33.42	141.70 ± 21.32	−11.23 (−25.95; 3.50)	0.129
VFDV right e	126.86 ± 37.14	145.48 ± 26.72	−18.61 (−35.06; −2.17)	0.028
VFDV left e	138.63 ± 32.71	147.44 ± 23.71	−8.80 (−23.29; 5.69)	0.222
VFDV right i	126.94 ± 33.45	146.73 ± 25.61	−19.79 (−34.64; −4.93)	0.011
VFDV left i	141.17 ± 27.18	151.46 ± 21.75	−10.28 (−22.38; 1.81)	0.092
3–5 weeks after surgery				
VFDV right a	135.48 ± 26.14	149.25 ± 21.63	−13.78 (−25.75; −1.80)	0.026
VFDV left a	142.17 ± 32.84	150.49 ± 19.23	−8.32 (−22.94; 6.31)	0.253
VFDV right e	146.09 ± 24.28	152.83 ± 25.45	−6.75 (−18.24; 4.75)	0.241
VFDV left e	145.20 ± 35.35	152.59 ± 18.88	−7.39 (−23.06; 8.27)	0.340
VFDV right i	157.09 ± 20.97	158.85 ± 20.78	−1.77 (−11.60; 8.07)	0.717
VFDV left i	146.29 ± 33.29	161.37 ± 19.01	−15.08 (−29.88; −0.28)	0.046
Change (1–3 days vs. baseline)				
VFDV right a	−16.79 ± 36.05	−1.93 ± 27.09	−14.85 (−30.85; 1.14)	0.067
VFDV left a	−8.50 ± 40.48	−0.63 ± 26.18	−7.87 (−25.71; 9.98)	0.372
VFDV right e	−24.88 ± 33.50	−1.66 ± 30.11	−23.21 (−38.24; 8.19)	0.004
VFDV left e	−9.04 ± 35.00	−0.60 ± 28.47	−8.44 (−24.04; 7.16)	0.276
VFDV right i	−24.45 ± 40.71	−3.39 ± 27.47	−21.06 (−39.03; −3.08)	0.024
VFDV left i	−11.97 ± 25.96	0.04 ± 24.84	−12.01 (−23.71; −0.31)	0.045
Change (3–5 weeks vs. baseline)				
VFDV right a	−1.71 ± 27.10	6.59 ± 29.69	−8.30 (−21.24; 4.64)	0.202
VFDV left a	3.20 ± 41.68	5.74 ± 27.40	−2.55 (−21.25; 16.16)	0.782
VFDV right e	−5.65 ± 30.44	3.95 ± 30.97	−9.60 (−23.95; 4.75)	0.183
VFDV left e	−2.48 ± 46.88	2.45 ± 26.43	−4.93 (−25.76; 15.91)	0.631
VFDV right i	5.70 ± 30.97	7.28 ± 29.13	−1.58 (−16.01; 12.85)	0.825
VFDV left i	−6.85 ± 43.03	7.92 ± 26.82	−14.77 (−34.01; 4.47)	0.127

Data presented as mean ± SD. MD—mean/median difference calculated as dysfunction group minus healthy group; CI—confidence interval; VFDF—vocal folds displacement velocity. Groups compared with Student’s *t*-test.

**Table 5:** Validity analysis of TLUS vs. laryngoscopy.

	Result				Sensitivity (95% CI)	Specifity (95% CI)	PPV (95% CI)	NPV (95% CI)	Accuracy (95% CI)
	TP	TN	FP	FN					
1–3 Days									
Total group	206	20	0	4	98.1 (95.2–99.5)	100.0 (83.2–100.0)	100.0	83.3 (65.5–92.7)	98.3 (95.6–99.5)
Female	163	15	0	3	98.2 (94.8–99.6)	100.0 (78.2–100.0)	100.0	83.3 (61.9–93.9)	98.3 (95.2–99.7)
Male	43	5	0	1	97.7 (87.9–99.9)	100.0 (47.8–100.0)	100.0	83.3 (41.9–97.2)	97.9 (89.2–99.9)
BMI < median (27.7)	105	9	0	1	99.1 (94.9–99.9)	100.0 (66.4–100.0)	100.0	90.0 (56.1–98.4)	99.1 (95.3–99.9)
BMI ≥ median (27.7)	101	11	0	3	97.1 (91.8–99.4)	100.0 (71.5–100.0)	100.0	78.6 (54.6–91.8)	97.4 (92.6–99.5)
Nonoverweight	72	5	0	1	98.6 (92.6–99.9)	100.0 (47.8–100.0)	100.0	83.3 (41.7–97.2)	98.7 (93.1–99.9)
Overweight	68	7	0	1	98.6 (92.2–99.9)	100.0 (59.0–100.0)	100.0	87.5 (50.0–98.0)	98.7 (92.9–99.9)
Obese	66	8	0	2	97.1 (89.8–99.6)	100.0 (63.1–100.0)	100.0	80.0 (50.5–94.0)	97.4 (90.8–99.7)
Age < median (46)	99	10	0	3	97.1 (91.6–99.4)	100.0 (69.2–100.0)	100.0	76.9 (52.2–91.0)	97.3 (92.4–99.4)
Age ≥ median (46)	106	10	0	1	99.1 (94.9–99.9)	100.0 (69.2–100.0)	100.0	90.9 (58.7–98.6)	99.2 (95.3–99.9)

TP—true-positive; TN—true negative; FP—false positive; FN—false negative; PPV—positive predictive value; NPV—negative predictive value; CI—confidence interval; BMI—Body Mass Index.

**Table 6:** Comparison of patients with visible and not visible vocal folds (before surgery).

Variable	Not Visible Vocal Folds	Visible Vocal Folds	RR/MD (95% CI)	*p*
Number of patients/surgeries	13/13	205/216		
Sex, female, *n* (%)	1 (7.7)	168 (82.0)	0.09 (0.01; 0.62)	<0.001
BMI, mean ± SD	32.34 ± 6.36	27.65 ± 6.10	4.69 (0.79; 8.60)	0.022
Smoking, *n* (%)	6 (46.2)	30 (14.6)	3.15 (1.61; 6.19)	0.003
Hemithyroidectomy	3 (23.1)	120 (55.6)	0.42 (0.04; 0.97)	0.041
Secondary surgery, *n* (%)	3 (23.1)	14 (6.5)	3.56 (1.17; 10.85)	0.027
Lateral lymph nodes, *n* (%)	2 (15.4)	2 (0.9)	16.60 (2.54; 108.71)	0.017
Bilateral RLN at risk				
T, *n* (%)				
1a	3 (37.5)	55 (43.3)	-	0.014
1b	0 (0.0)	38 (29.9)		
2	4 (50.0)	16 (12.6)		
3a	0 (0.0)	15 (11.8)		
3b	1 (12.5)	2 (1.6)		
4a	0 (0.0)	1 (0.8)		
Multifocal, *n* (%)	4 (30.8)	22 (10.2)	3.02 (1.22; 7.48)	0.046
Left lobe volume, cm^3^, median (Q1; Q3)	10.60 (7.89; 15.89)	6.10 (3.91; 10.10)	4.50 (0.30; 8.46)	0.034
Lateral approach when anterior impossible, *n* (%)	5 (38.5)	16 (7.5)	5.14 (2.24; 11.84)	0.003
Visible true vocal folds, *n* (%)	0 (0.0)	53 (24.5)	-	0.043
fT3	3.69 (3.55, 4.02)	3.24 (2.96, 3.58)	0.46 (0.27; 0.75)	<0.001

Data presented as *n* (%) for nominal variables and as mean ± SD or median (Q1; Q3) for continuous variables, depending on the normality of the distribution. BMI—body mass index; CI—confidence interval; MD—mean/median difference calculated as paralysis group minus healthy group; Q—quartile; RR—risk ratio. Groups were compared with the chi-square test or Fisher’s exact test for nominal variables and with Student’s *t*-test or Mann–Whitney U test for continuous variables.

**Table 7:** Comparison of dysfunction and healthy cases 1–3 days after surgery.

Variable	Dysfunction	Healthy	RR/MD (95% CI)	*p*
Number of patients/surgeries	23/24	196/206		
TLUS data Visible vocal folds	23 (95.8)	192 (93.2)	1.03 (0.94; 1.13)	>0.999
Normal vocal fold movement	3 (13.0)	192 (100.0)	-	<0.001
USG vocal fold symmetry still	3 (13.0)	191 (99.5)	0.13 (0.05; 0.38)	<0.001
Symmetry during cough	3 (13.0)	191 (99.5)	0.13 (0.05; 0.38)	<0.001
Symmetry during phonation	3 (13.0)	191 (99.5)	0.13 (0.05; 0.38)	<0.001
Symmetry during swallowing	3 (13.0)	191 (99.5)	0.13 (0.05; 0.38)	<0.001
Arytenoid symetry	3 (13.0)	191 (99.5)	0.13 (0.05; 0.38)	<0.001
Crescendo–decresceno wave Clinical findings	4 (17.4)	0 (0.0)	-	<0.001
Hoarseness	21 (87.5)	36 (17.6)	4.96 (3.55, 6.92)	<0.001
Problems with high pitch	17 (70.8)	20 (9.8)	7.23 (4.43, 11.78)	<0.001
Dysphagia	2 (8.3)	2 (1.0)	8.50 (1.25, 57.62)	0.056

MD—mean/median difference calculated as paralysis group minus healthy group; RR—risk ratio.

## Data Availability

The authors confirm that the data supporting the findings of this study are available in supplementary materials.

## Supplementary Materials

The following are available online at https://www.mdpi.com/article/10.3390/jcm11061691/s1, Table S1: Cases characteristics, Figure S1. Type of surgery (*n* = 230); Figure S2. The effect of sex on patient’s vocal folds visibility (before surgery); Figure S3. The effect of weight on patient’s vocal folds visibility (before surgery).
