# Supplementary material for: Application of Translaryngeal Ultrasound (TLUS) in Patients with Neck Surgery—A Single-Centre, Prospective Cohort Study on Technique Evaluation
Source: J Clin Med. 2022 Mar 18;11(6):1691. doi: 10.3390/jcm11061691 (PMC8953745; doi:10.3390/jcm11061691)
Supplement: Supplementary file 1 [file jcm-11-01691-s001.zip › jcm-1614570-supplementary.pdf]

Table S1. Cases characteristics

Variable	N	Level
Number of surgeries	230	
Dysfunction, n (% of group) / % of all dysfunctions		24 (10.4) / 100.0
Paresis		8 (3.5) / 33.3
Left side		3 (1.3) / 12.5
Right side		5 (2.2) / 20.8
Paralysis		16 (7.0) / 66.7
Left side	230	10 (4.3) / 41.7
Right side		6 (2.6) / 25.0
Transient		20 (9.0) / 84.0
Permanent		4 (1.7) / 16.7
Permanent – paresis		3 (1.3) / 12.5
Permanent – paralysis		1 (0.4) / 4.2
Reoperation, n (%)	230	14 (6.1)
BACC, n (%)		
1		1 (0.5)
2		19 (9.9)
3		14 (7.3)
4		40 (20.8)
5	192	46 (24.0)
6		39 (20.3)
7 – parathyroid		17 (8.9)
8 – local cancer recurrence or lymph nodes metastases		16 (8.3)
Cancerous indication, n (%)		
Yes	230	196 (85.2)
No		34 (14.8)
Thyroid or parathyroid, n (%)		
Carcinoma		196 (85.2)
Benign thyroid disease	230	18 (7.8)
Parathyroid		19 (8.3)
Type of surgery, n (%)		

Variable	N	Level
Thyroidectomy with central	230	58 (25.2)
Hemithyroidectomy with central		124 (53.9)
Thyroidectomy with lateral neck dissection		15 (6.5)
Lymph nodes metastatic		12 (5.2)
Surgery of recurrent disease in postoperative bed		5 (2.2)
Isthmectomy		3 (1.3)
Parathyroid		19 (8.3)
Trachea release		1 (0.4)
Secondary surgery, n (%)	230	17 (7.4)
Lateral lymph nodes, n (%)		
Bilateral RLN at risk	230	4 (1.7)
Unilateral RLN		9 (3.9)
Side, n (%)		
Left	121	63 (52.1)
Right		58 (47.9)
Radicalisation, n (%)	228	46 (20.2)
Size of tumor, mm, mean±SD		
Diameter 1	189	17.46±13.49
Diameter 2	189	14.76±11.16
Diameter 3	189	22.28±16.92
Max diameter	189	22.50±16.89
Tumor volume, cm^3, median (Q1;Q3)	189	1.04 (0.23;5.51)
Size of tumor path, mm, mean±SD	181	19.22±17.11
Lesion location, n (%)		
Left	215	72 (33.5)
Right		86 (40.0)
Both		16 (7.5)
Parathyroid		20 (9.3)
Isthmus		9 (4.2)
Lymph node		13 (6.0)
T, n (%)		

Variable	N	Level
1a	136	58 (42.6)
1b		39 (28.7)
2		20 (14.7)
3a		15 (11.0)
3b		3 (2.2)
4a		1 (0.7)
Multifocal, n (%)	230	26 (11.3)
Lymph nodes metastases, n (%)	230	36 (15.7)
Lateral		21 (9.1)
Central		16 (7.0)
N, n (%)	129	
0		93 (72.1)
1a		15 (11.6)
1b		21 (16.3)
Cancer / benign, n (%)	230	
Benign t		80 (34.8)
Cancer		150 (65.2)
Type of cancer, n (%)	168	
Papillary		102 (60.7)
Folliculary		11 (6.5)
Medullary		16 (9.5)
Hurthle		4 (2.4)
Anaplastic		1 (0.6)
Other cancer		4 (2.4)
Parathyroid		19 (11.3)
Border-line tumors group 1		11 (6.5)
Previous neck radiation, n (%)	229	-
Previous neck surgery, n (%)	229	62 (27.1)
Right lobe width, mm, mean±SD	223	17.30±10.19
Right lobe height, mm, mean±SD	223	17.25±9.61
Right lobe length, mm, mean±SD	223	46.00±21.89

Variable	N	Level
Right lobe volume, cm^3, median (Q1;Q3)	223	8.51 (5.18;12.08)
Left lobe width, mm, mean±SD	222	17.25±10.40
Left lobe height, mm, mean±SD	222	16.45±9.74
Left lobe length, mm, mean±SD	222	46.59±20.22
Left lobe volume, cm^3, median (Q1;Q3)	222	6.34 (3.95;10.56)
Thyroid volume, cm^3, median (Q1;Q3)	222	15.73 (9.12;26.60)
Echogenicity homogenous, n (%)	220	100 (45.5)
Retrosternal thyroid, n (%)	227	24 (10.6)
Previous hoarseness, n (%)	229	4 (1.7)
Lateral approach when anterir imposible, n (%)	228	21 (9.2)
Visible true vocal folds, n (%)	227	53 (23.3)
Before surgery		
Singal Amplitude – left	131	0.74 (0.34;1.20)
Latency – left	131	6.20 (5.80;6.75)
Singal Amplitude – right	135	0.83 (0.38;1.57)
Latency – right	135	3.85 (3.60;4.23)
After surgery		
Singal Amplitude – left	129	0.53 (0.24;0.94)
Latency – left	129	6.40 (6.00;6.95)
Singal Amplitude – right	136	0.72 (0.28;1.20)
Latency – right	136	4.00 (3.75;4.35)
Change		
Singal Amplitude – left	117	-0.08 (-0.41;0.11)
Latency – left	128	0.10 (-0.15;0.55)
Singal Amplitude – right	117	-0.08 (-0.47;0.14)
Latency – right	128	0.15 (-0.10;0.40)
RLN entrapment to tumor, n (%)	22	7 (31.8)
WBC	230	6.73±2.21
TSH	228	1.52 (0.84;2.23)
fT3	229	3.25 (2.97;3.63)
fT4	229	1.37±0.34

Variable	N	Level
Ca	230	2.45±0.19
Ca after	230	2.17±0.14
Unpleasant USG, 0-10 scale, median (Q1;Q3)	230	0.00 (0.00;1.00)
Unpleasant laryngoscopy, 0-10 scale, median (Q1;Q3)	230	2.00 (1.00;5.00)
USG vs. laryngoscopy 1st assessment, n (%)		
More unpleasant laryngoscopy		150 (65.2)
More unpleasant USG	230	10 (4.3)
Both the same		70 (30.4)
USG vs. laryngoscopy 2nd assessment, n (%)		
More unpleasant laryngoscopy		32 (66.7)
More unpleasant USG	48	5 (10.4)
Both the same		11 (22.9)

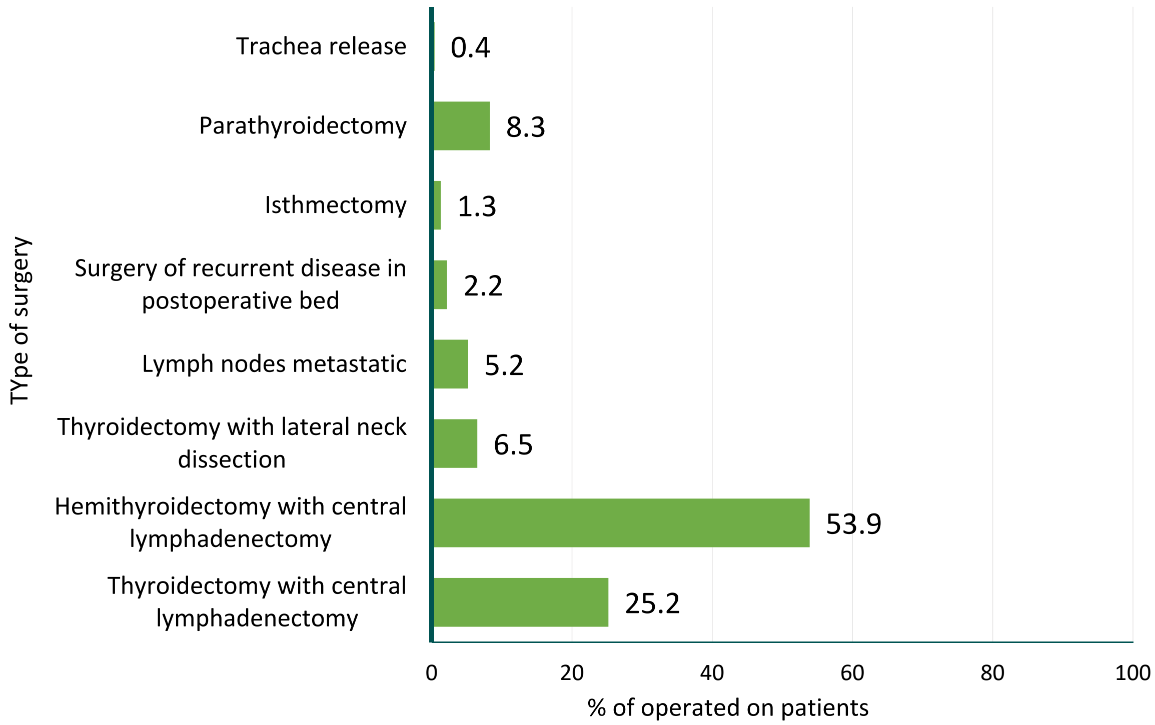

Figure S1. Type of surgery (n=230).

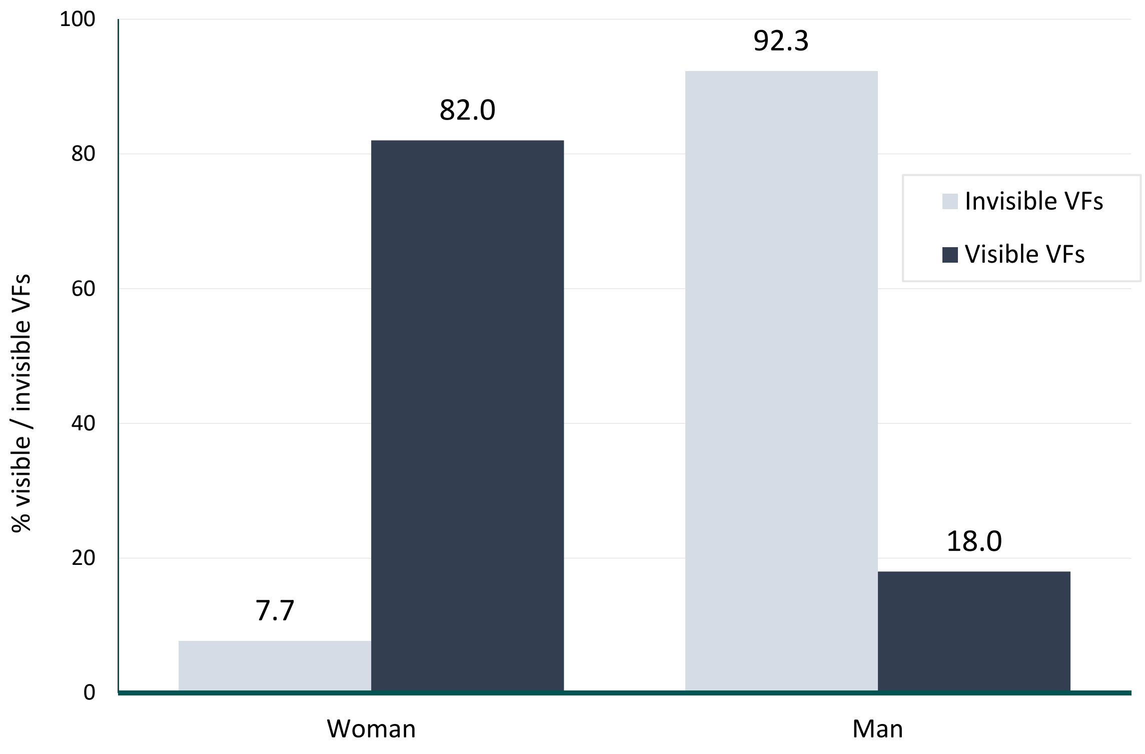

Figure S2. The effect of sex on patient’s vocal folds visibility (before surgery).

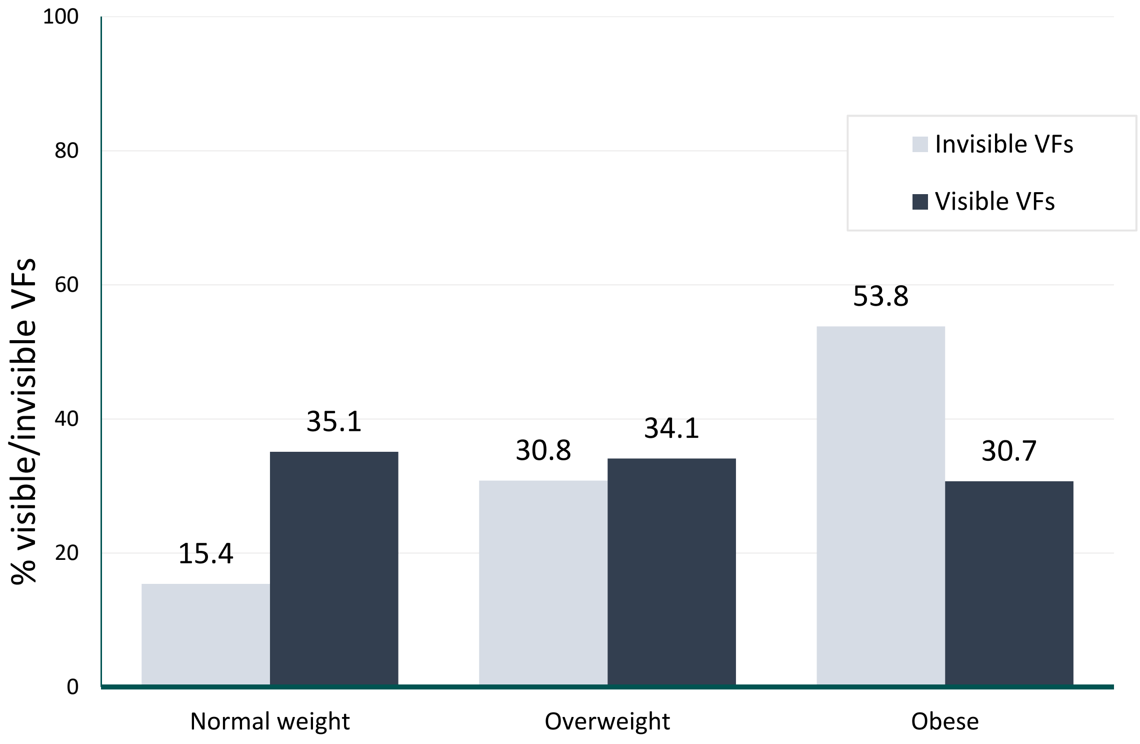

Figure S3. The effect of weight on patient’s vocal folds visibility (before surgery).
